# Microbiology sampling in non-cystic fibrosis bronchiectasis cases from northern Alberta

**DOI:** 10.1371/journal.pone.0288704

**Published:** 2023-07-14

**Authors:** Mitchell J. Wagner, Monette Dimitrov, Grace Y. Lam, Winnie Leung, Gregory J. Tyrrell, Dilini Vethanayagam

**Affiliations:** 1 Faculty of Medicine and Dentistry, University of Alberta, Edmonton, Alberta, Canada; 2 Department of Medicine, Faculty of Medicine and Dentistry, University of Alberta, Edmonton, Alberta, Canada; 3 Provincial Laboratory for Public Health, Alberta Health Services, Edmonton, Alberta, Canada; Laurentian University, CANADA

## Abstract

Non-cystic fibrosis bronchiectasis (NCFB) is a chronic respiratory disease resulting in chronic cough, thick sputum, and lower airway microbial colonization, akin to patients with cystic fibrosis (CF). NCFB is a common, yet under recognized entity which inflicts significant morbidity and mortality particularly to older individuals, with a rising prevalence in the developed world. Given that sputum cultures are a non-invasive method to characterize the lower airway microbiota in NCFB patients, for which pathogenic organisms are associated with worsened outcomes, we sought to characterize the microbiological pattern and clinical outcomes associated with sputum culture in a cohort of NCFB patients from Western Canada. A total of 20 subjects were prospectively recruited from various bronchiectasis clinics across the Greater Edmonton area. A retrospective chart review and a symptoms questionnaire was performed, gathering information not limited to symptoms, comorbidities, exacerbations, hospitalizations, sputum production, and sputum culture results over the prior 5 years. Subjects reported frequent hospitalization alongside a significant burden of symptoms. A large majority of sputum cultures grew pathogenic organisms such as *Haemophilus influenzae* and *Pseudomonas aeruginosa*. We also note the considerable waste and inefficiency associated with sputum cultures, outlining areas for which this important diagnostic modality can be improved. Accurate characterization of the airway microbiota alongside efficient delivery of health services are key to ensuring the proper treatment of individuals with NCFB, given their high disease burden and frequent hospitalization.

## Introduction

Non-cystic fibrosis bronchiectasis (NCFB) is a chronic disease of the airways characterized by abnormally and permanently dilated airways [1, 2]. Patients afflicted by NCFB most commonly suffer from recurrent infections, a persistent and productive cough, inflammation, fatigue, and shortness of breath [1–4]. Currently, the disease cannot be cured but effective management of this chronic disease can impact quality of life [3, 5]. Initially, it was thought that NCFB was uncommon, being regarded as an ‘orphan disease’. However, as diagnostic techniques for NCFB improve, the disease has gained increasing attention in the developed world as its prevalence continues to grow, especially in older populations [2, 3]. A cohort study of bronchiectasis patients found that in the United Kingdom between 2004 to 2013 the prevalence of NCFB in both men and women rose over time, approaching 300–550 individuals per 100,000 population suffering from bronchiectasis [6]. Similarly, a study in the United States estimated prevalence over 2000–2007 to be 1106 cases per 100,000 people over the age of 65, and increasing at a rate of 8.7% per year [7]. Thus, NCFB represents a considerable source of disease burden amongst older populations and the general population that seems to be increasing.

Given that lower respiratory tract infections are common in patients suffering from NCFB and sputum is often produced, sputum culture is a non-invasive way to monitor microbiological progression and the presence of key pathogens [1, 8]. Most commonly, *Pseudomonas aeruginosa*, *Staphylococcus aureus*, *Haemophilus influenzae* and non-tuberculous mycobacteria are pathogens found in the lungs of those with NCFB [3, 4]. *P*. *aeruginosa* and *H*. *influenzae* are particularly associated with worsened clinical outcomes, such as lung function decline and increased morbidity and mortality, emphasizing the importance of regular sputum collection with microbiological workup [9, 10]. A meta-analysis conducted in 2015 of culture from sputum or bronchoalveolar lavage fluid (BALF) showed that in comparison, sputum culture more often identified *P*. *aeruginosa* [11]. Given this pathogen’s association to worsened outcomes in NCFB patients, it is important that diagnostic modalities can sensitively assess its presence. More broadly speaking, knowledge of the microbiological landscape within the lung is critical for tailoring antibiotic choice and verifying eradication efforts of specific pathogens in NCFB patients, and reliable monitoring of such landscapes is imperative to support efforts in antibiotic stewardship [9, 12]. Collection of expectorated sputum is also a readily available as a diagnostic and monitoring tool, and quality sampling should be achievable with relatively little staff training [8, 13, 14].

Culture of sputum samples has been a mainstay method of assessing potential pathogens within the airway microbiota. Although, culture is known to select for the growth of known pathogens, which has prompted investigation using next generation sequencing (NGS) techniques that don’t suffer this limitation [15]. Recent studies have demonstrated that NGS can be of utility in the assessment of the airway microbiota in NCFB patients and microbial diversity may represent a biomarker for disease states, though is relatively underexplored in the context of NCFB [10, 15]. Woo *et al*. found in a longitudinal analysis of the 16S rRNA gene of bacteria from NCFB patients that those with declining forced expiratory volume in one second (FEV_1_) had a lower alpha diversity, though no associations could be drawn between any type of bacteria and an observation of decreasing FEV_1_. [10]. Another longitudinal analysis by Cox *et al*. showed that while there was a lack of association between clinical state and any single bacteria, 16S rRNA sequencing was able to detect *P*. *aeruginosa* and *H*. *influenzae* and especially *Streptococcus pneumoniae* in many instances where sputum culture did not detect these pathogens [16]. In comparison to sputum culture, NGS techniques like 16S rRNA sequencing reflects an interesting avenue to perhaps discover new insights into the role of microbial diversity in NCFB patients, however further research is needed to determine whether NGS can provide robust biomarkers of disease progression. Studies have shown similar microbial profiles from sputum assessment to sputum culture [17], and as it stands, the method is significantly less ubiquitous amongst lab facilities compared to sputum culture; therefore, efforts that maximize the utility of sputum culture are perhaps currently of greater importance to patient treatment and resource optimization.

Despite its upsides, the use of sputum cultures has yet been criticized due to its low specificity and sensitivity [13, 14, 18]. A lack of influence on clinical decision making in the context of pneumonia has also been noted [13, 19]. A lowering of confidence in the use of sputum culture for clinical decision making is perhaps due to the low yield of sputum cultures and a relatively low proportion of quality samples subject to analysis; it is estimated that only 25–60% of obtained sputum samples are high quality, heavily dependent on collection quality but also influenced by variable transport times, antibiotic use, and processing practices [13, 14]. A further 30–40% of good quality specimens will fail to grow pathogens [4]. Standardized policies and protocols regarding sample collection by staff, and initial sample screening, specimen plating and duration of plating times by clinical microbiology laboratories can potentially lead to improved detection rates of pathogenic microbes within the lower airways while saving costs [8, 13].

Given the utility of sputum culture yet low yields, there exists a need for quality assurance and standardization of sputum analysis laboratory practices for NCFB with other respiratory diseases. Though many studies have analyzed sputum cultures to evaluate the airway of NCFB patients [11, 20, 21], few have provided commentary on areas of quality improvement for sputum culture which would make the modality more robust [13]. Thus, we sought to recruit a cohort of NCFB patients to characterize short and long-term clinical outcomes alongside sputum culture processes at our local institutions in hopes of identifying potential areas for quality improvement. We outline a number of areas that could be improved upon.

## Methods

Our study was approved by our institutional ethics board (University of Alberta Health Research Ethics Board, study ID Pro00049402 (S2 File)). To be included in the study, subjects were required to be over the age of 18 and have been diagnosed with non-cystic fibrosis bronchiectasis. Participants were prospectively recruited from various primary care and specialty clinics in the Greater Edmonton area, in the province of Alberta, Canada, in the summer of 2015. Given the difficulty in recruiting a sizeable population of individuals with bronchiectasis, we elected to recruit through physician offices *via* convenience sampling. Each participant in the study provided written consent by way of a patient information consent form (S1 File). This allowed participants to submit to a questionnaire which collected data regarding demographics as well as their self-reported symptoms, disease duration, sputum collection procedure, treatment, and co-morbidities over the previous year and last 5 years from the date of recruitment. Additionally, a chart review of electronic healthcare records was conducted by author MD to verify self-reported data, and collect data on expectorated sputum culture results, associated diagnostic imaging, and emergency visits. Patients who were unable to complete the questionnaire or were missing key data regarding sputum culture results were excluded from the study. All collected information was de-identified before analysis, conducted from May to August 2015. Processes for sputum sample collection and analysis (specifically antimicrobial susceptibility testing (AST), Q-scoring, organism identification) were also reviewed to evaluate variability in NCFB sputum sample microbiological workup.

A study size of 20 participants was arrived at based on patient availability and clinic participation. As the objective of our study was to characterize the burden of symptoms and sputum assessment in a local cohort of NCFB patients, no statistical analysis was conducted due to the lack of a comparator group. Data from the questionnaire and retrospective chart review was reported as proportions.

## Results

A total of 20 subjects were recruited. As summarized by Table 1, most subjects were female (13/20, 65%) and Caucasian (16/20, 80%). The mean age of the cohort was 60 years. The mean self-reported age of bronchiectasis diagnosis was 46 years (median 53 years). The cohort presented with a variety of comorbidities, asthma being the most common (50%) (Table 1).

**Table 1:** Patient information as collected by survey.

	Patient demographics	Proportion of patients self-reporting (%)
***Sex***	Male	7/20 (35%)
	Female	13/20 (65%)
***Ethnicity***	Caucasian	16/20 (80%)
	Asian	2/20 (10%)
	Metis	2/20 (10%)
	Other	1/20 (5%)
***Age of diagnosis of bronchiectasis***	<30 years	5/20 (25%)
	31–50 years	4/20 (20%)
	51–70 years	10/20 (50%)
	>71 years	1/20 (5%)
***Years since diagnosis of bronchiectasis***	0–5 years	12/20 (60%)
	6–10 years	0/20 (0%)
	11–20 years	2/20 (10%)
	>21 years	5/20 (25%)
	Unsure	1/20 (5%)
***Reported cause of bronchiectasis***	Pneumonia	12/20 (60%)
	Childhood pneumonia	3/20 (15%)
	Immune deficiency	6/20 (30%)
	Pertussis/whooping cough	5/20 (25%)
	Non-tuberculosis mycobacteria	5/20 (25%)
	Tuberculosis	1/20 (5%)
	Primary ciliary dyskinesia	1/20 (5%)
	Other	4/20 (20%)
**Comorbidities**	Asthma	10/20 (50%)
	COPD	7/20 (35%)
	Hypertension	7/20 (35%)
	Diabetes	1/20 (5%)
	Cancer	3/20 (15%)
	Stroke/TIA	1/20 (5%)
	Skin Lesions	6/20 (30%)
	Anxiety/Depression	4/20 (20%)
	Gastric Reflux	4/20 (20%)

The cohort reported typical NCFB symptoms: coughing (95%), sputum production (90%), dyspnea (80%), fatigue (80%), hemoptysis (60%), chest pain (50%), wheezing (45%), poor appetite (40%), and night sweats (30%). Sputum expectoration was common (80%), varying in color and amount (Table 2). Most patients reported being hospitalized due to their bronchiectasis at least once over the past 5 years (80%), with 65% in the last year. Many subjects had chest CT (95%), sputum cultures (75%), and bronchoscopy (70%). Of patients subject to sputum collection, 65% reported not being taught proper sputum collection technique, and 45% of patients collected sputum on their own for delivery to a specimen collection site before transport to a microbiology laboratory.

**Table 2:** Frequency of specific symptoms experienced by NCFB patients via survey.

	Type of symptom	Proportion of patients self-reporting (%)
**Lung symptoms:**	Coughing	19/20 (95%)
	Sputum production	18/20 (90%)
	Shortness of breath	16/20 (80%)
	Hemoptysis	12/20 (60%)
	Chest pain/tightness	10/20 (50%)
**General Symptoms:**	Wheeze	9/20 (45%)
	Fatigue	16/20 (80%)
	Poor appetite	8/20 (40%)
	Unexplained weight loss	8/20 (40%)
	Night sweats	6/20 (30%)
**Sputum expectoration:**	1 tsp to ½ cup a day on most days	9/20 (45%)
	1 tsp or less a day on most days	7/20 (35%)
	Don’t cough up sputum regularly	4/20 (20%)
**Sputum color:**	Yellow	8/20 (40%)
	Mixed	7/20 (35%)
	Green	2/20 (10%)
	No color	2/20 (10%)
	Brown	1/20 (5%)

Antibiotic use was prevalent in the cohort, with 80% reporting having used antibiotics in the previous year and 90% in the last 5 years. Reported antibiotic use varied from rotating between classes (60%), continuously (35%), and intermittently (55%).

Exacerbations were common, with 65% of patients reporting ≥1 exacerbation in the past year, 10% for each of the past 1–2 years and 3–5 years, 5% >5 years ago, and 10% never experiencing an exacerbation. Of those experiencing exacerbations, between 3–6 exacerbations per year was most common (45%), followed by 1–2 per year (33%), with >6 or <1 exacerbations per year being least common (11% each).

Over the prior 5 years from time of subject recruitment, a total 128 sputum samples were submitted to two laboratories, with 108 (84%) being acceptable for microbiological analysis. Out of the 108 analyzed samples, 81 cultures (75%) grew typical NCFB pathogens, including *S*. *aureus* (9.3%), *H*. *influenzae* (13%), *Streptococcus pneumoniae* (5.6%) and *P*. *aeruginosa* (65%). A similar breakdown has been observed over the past year (Table 3). 23 cultures (21.3%) grew no pathogens despite previous cultures positive for NCFB pathogens, and 4 cultures (3.7%) grew no pathogens with previous negative cultures.

**Table 3:** Frequency of microbial detection from sputum cultures over the prior 12 months (1 year) and 60 months (5 years) in NCFB patients.

Organism	Frequency of detection in the past 12 months (1 year) (%)	Frequency of detection in past 60 months (5 years) (%)
*Pseudomonas aeruginosa*	13/28 (46.4%)	70/108 (64.8%)
Nonmucoid	10/28 (35.7%)	38/108 (35.2%)
Mucoid	3/28 (10.7%)	20/108 (18.5%)
Unspecified	0	12/108 (11.1%)
*Candida spp*.	7/28 (25.0%)	17/108 (15.7%)
*Haemophilus influenzae*	2/28 (7.1%)	14/108 (13%)
*Staphylococcus aureus*	2/28 (7.1%)	10/108 (9.3%)
*Streptococcus pneumoniae*	1/28 (3.6%)	6/108 (5.6%)
*Stenotrophomonas maltophilia*	0	3/108 (2.8%)
*Pseudomonas putida*	2/28 (7.1%)	2/108 (1.9%)
*Moraxella*	0	2/108 (1.9%)
*Pseudomonas fluorescens*	0	1/108 (0.9%)
*Streptococcus pyogenes*	0	1/108 (0.9%)
*Escherichia coli*	1/28 (3.6%)	1/108 (0.9%)
*Enterococcus faecalis*	1/28 (3.6%)	1/108 (0.9%)
Gram negative bacilli	1/28 (3.6%)	0

We also had the opportunity to review the requests written on sputum sample microbiology requisitions, which influence treatment of the samples within the laboratory. Out of 108 cultures performed, only 9 sputum requisitions from 9 patients mentioned “bronchiectasis”. These requisitions were amongst others incorrectly designated as “CF sputa”, “chronic pseudomonas”, and “respiratory cultures”. Interestingly, requisitions requesting AST trended toward a greater proportion of cultures positive for NCFB pathogens (Tables 4 and 5).

**Table 4:** Different requisition requests with associated frequency of at least one pathogen grown over the past 60 months (5 years).

Request written on sputum microbiology requisition	Proportion of pathogens grown per requisition
“Bronchiectasis” (4)	1/4 (25%)
“Bronchiectasis with antibiotics testing” (5)	4/5 (80%)
“Respiratory cultures with antibiotics testing”	39/39 (100%)
“Respiratory cultures” (9)	6/9 (66.6%)
“CF sputa” (1)	1/1 (100%)
“Chronic Pseudomonas” (1)	1/1 (100%)
“Respiratory cultures with antibiotics testing and COPD and shortness of breath” (3)	3/3 (100%)
“Respiratory cultures with *Nocardia*” (2)	1/2 (50%)

**Table 5:** Different requisition requests with associated frequency of at least a single pathogen grown over the past 12 months (1 year).

Request written on sputum microbiology requisition	Pathogens grown
“Bronchiectasis” (1)	None (0/1)
“Bronchiectasis with antibiotics testing” (5)	4/5 (80%)
“Respiratory cultures with antibiotics testing” (3)	3/3 (100%)
“Respiratory cultures” (18)	13/18 (72.2%)
“CF sputa” (2)	2/2 (100%)
“Respiratory Cultures with antibiotics testing and pneumonia” (1)	1/1 (100%)

## Discussion

The objective of the study was to characterize the short- and long-term clinical outcomes and identify areas for quality improvement regarding sputum sampling and analysis in a local cohort of NCFB patients. Our cohort reported classical symptoms of NCFB patients [1, 9, 22, 23]: productive cough, fatigue, hemoptysis, dyspnea, wheezing, chest pain were exhibited by a majority of our cohort (Table 1). The heterogeneous etiology of NCFB was also exemplified by our cohort, with medical records showing a variety of causes for their NCFB (Table 2). Despite continuous or intermittent antibiotic use (>90% over the past 5 years), our cohort reported by survey considerable exacerbations, hospitalizations, and general disease burden regardless. Over the past 5 years, almost 65% of sputum cultures identified *P*. *aeruginosa* with a smaller amount identifying *H*. *influenzae* (13%). Their frequency of detection decreased to 46% and 7.1% respectively over the past year. A similar study which analyzed the proportions of pathogens isolated during sputum culture found instead that *H*. *influenzae* was the most common pathogen at 47%, followed by *P*. *aeruginosa* at 18%. However, patients within their cohort were those in a stable state and were not on antibiotics for at least one month before being recruited [24]. The U.S Bronchiectasis Research Registry reported that out of 1826 patients with NCFB, the most common pathogens found were instead *P*. *aeruginosa* (33%) and *S*. *aureus* (12%) [3]. Antibiotic use targeting *H*. *influenzae* and failing to eradicate antibiotic resistant *P*. *aeruginosa* could account for the commonly observed representation of *P*. *aeruginosa* in our cohort and the U.S Bronchiectasis registry, compared with that of King *et al*. [24].

The persistence of pathogens (particularly *H*. *influenzae* and *P*. *aeruginosa*) within sputum cultures over 5 years in combination with reportedly widespread antibiotic use in our cohort could exemplify the ineffectiveness of antibiotic therapy at limiting these pathogens within the airway, and could suggest that efforts to limit antibiotic resistance in the context of NCFB are failing [25]. Almost 25% of *P*. *aeruginosa* isolates were of the mucoid phenotype, which is known to be indicative of antimicrobial resistance [26]. Given that these isolates, especially mucoid *P*. *aeruginosa*, have been associated with decreased lung function and worsened quality of life in bronchiectasis patients [4, 12, 25, 26], existing methods of management and therapy for these patients could be reviewed for improvement, and provide a better quality of life. Indeed, the prospect of anti-microbial stewardship is of great importance especially with regard to *P*. *aeruginosa* in NCFB. In a study of sputum culture by Gao *et al*. in 2018, patients with antimicrobial resistant *P*. *aeruginosa* had been on antibiotics for a longer period of time before the study and were subject to a greater amount of hospital admissions and acute exacerbations compared to patients without resistant *P*. *aeruginosa* [27]. Therefore, avoidance of ineffective antibiotic therapy that may generate antibiotic resistant strains of *P*. *aeruginosa* could decrease morbidity in NCFB patients. This could indeed be aided by improving the utility of sputum culture, a key tool in monitoring the airway microbiome, as key isolates and their antibiotic resistance can be measured.

Indeed, our study uncovered a few areas of potential improvement with sputum sampling and analysis at our local institution. First, a significant proportion (65%) of our cohort did not recall being taught contamination-minimizing sputum collection and handling/transport techniques. This is reinforced by our finding that 20/128 (16%) of cultures had to be discarded due to oropharyngeal contamination representing a considerable waste of time and resources on part of clinical and laboratory staff. Sputum samples can be hard to produce for some individuals (20% of our cohort did not produce sputum regularly), therefore wasted collections can be concerning as timing may be critical to management, especially for tailoring antimicrobial choice. It can also be a risk that clinical decisions may be made off of inappropriate sputum samples if laboratories fail to perform quality checks of the submitted sputum [13]. Education of nursing staff who collect sputum samples from patients has been shown to be an effective strategy in this regard. Moncayo-Nieto and colleagues implemented a staff education intervention and found that the amount of appropriate sputum samples for analysis significantly improved from 39% to 60%, with significantly less samples submitted despite identical analysis periods [13]. The proportion of acceptable sputum samples from culture in our study (84%) was higher than other studies [13, 19], however there is still room for improvement.

Second, delivery times are a variable in sputum processing that could use standardization. Results showed 23 of the 108 (21%) remaining sputum cultures did not grow pathogens, despite previous cultures with growths. Similar proportions of quality sputum isolates failing to grow pathogens have been reported [24]. While this could be explained by antimicrobial therapy or even the natural progression of the microbiome over time, it has also been noted that the recovery of pathogens like *H*. *influenzae* and *S*. *pneumoniae* is greatly improved if delivered to the microbiology laboratory within 3 hours of collection from the patient [28, 29]. The fact that 45% of our cohort reported delivering their own samples to a specimen collection site for subsequent delivery to a microbiology laboratory represents potential variability in delivery and processing times, which could influence pathogen detection during sputum culture. This could also account for the discrepancy between our study’s frequency of *H*. *influenzae* detection and other studies [24]. While we were unable to measure the time between collection and analysis, this can be assessed in future studies. Nonetheless, efforts should be made to standardize delivery times such that pathogen detection for sputum is maximized.

Third, analysis of microbiology requisition forms from NCFB sputum samples showed that over the last 5 years, a large proportion contained requests for processing as samples other than being bronchiectasis samples (Table 4). Requests written on these requisitions dictate how sputum samples are processed, influencing the pathogens grown. For example, at the time of this study in northern Alberta sputum samples needing a work-up for CF would be sent to the Alberta Provincial Lab (APL) (microbiology laboratory that serviced the northern Alberta CF clinics), whereas non-CF sputa were routed to nearby community or hospital laboratories for analysis, reflecting differing practices for sample workup. At community labs, non-CF specimens with good Q-score (minimal oropharyngeal contamination) are surveyed further for pathogens, however in the case of CF sputa at the APL and in published literature, specimens are processed regardless of Q-score [30]. A good Q-score based on gram-staining also determines whether antibiotics testing is done and whether the sample needs to be grown on other plates [31]. Though sample size was limited, referrals requesting this had a seemingly greater proportion of cultures positive for NCFB pathogens, which may reflect different handling that resulted in more pathogens grown. Therefore, requests should be accurate such that sputum samples undergo standard processing.

We also observed a similar microbiological profile from NCFB sputum samples to those found in CF patients (Table 3) [18], suggesting that NCFB and CF sputa samples should be processed similarly. Both NCFB and CF sputum culture involves incubation on MacConkey, blood, and chocolate agar for fastidious organisms. At the APL, CF sputum samples undergo additional culture on selective agars such as mannitol salt agar and *Burkholderia cepacia* selective agar [31], unlike NCFB sputum samples. In the Greater Edmonton area, mannitol plates for *S*. *aureus* are not utilized consistently for the culture of sputum samples. Given the fastidious nature of NCFB pathogens, further work is needed to evaluate processes to standardize microbiology laboratory processing for NCFB to possibly better align with CF sputa assessment, given that Q scores are utilized for detailed work up of NCFB samples, but not CF samples at our local institutions. Practices like Q-scoring are not utilized in all centers. perhaps in favor of other subjective evaluations [32, 33].

Our study has a handful of notable limitations. A large proportion of patients reported being on antibiotics, influencing the microbiota able to be assessed by sputum culture. The administration of a questionnaire to patients also introduced recall and reporting bias. We also were unable to collect information about which sputum samples were collected during exacerbations versus chronic stable states, during which the microbiota can be altered [34]. As mentioned, the sizeable proportion of patients who took their own sputum samples and delivered them themselves introduced variability, which may take away some generalizability of the results.

The current study characterizes the significant disease burden that afflicts patients with NCFB, as well as the microbiological composition of their airways as reflected by sputum culture over the past 5 years. Via our analysis of local sputum collection and culture protocols in our cohort, we propose several quality improvements and standardizations that can be applied to the collection and analysis of sputum cultures to improve its diagnostic and monitoring efficacy in the context of NCFB. Standardized patient education can be introduced to reduce the degree of improper samples and degree of oropharyngeal contamination. Delivery times (part of sample turn-around times) could be standardized by having patients collect sputum at the specimen collection centres rather than on their own. Correctly entering the necessary patient data by submitters on microbiology requisitions could be routinely audited to ensure correct labelling, preventing inappropriate workup by laboratory services. We also propose that the microbiological laboratory protocol used for processing NCFB sputum samples follow protocols used for processing CF sputum samples, given their similar microbiota. Clinicians who rely on these samples for clinical decision making could benefit from regular evaluation of these standards, with this practice perhaps being incorporated into accreditation of these facilities. Standardization between lab processes for sputum microbiology needs to be further evaluated as it could positively impact health care utilization and relevant clinical outcomes for all high-risk respiratory patients with underlying bronchiectasis of any cause.

## Supporting information

S1 Checklist Completed STROBE checklist. (DOCX)

S1 File Patient consent form. (DOCX)

S2 File Ethics approval document. (PDF)
