# Supplementary material for: Microbiology sampling in non-cystic fibrosis bronchiectasis cases from northern Alberta
Source: PLoS One. 2023 Jul 14;18(7):e0288704. doi: 10.1371/journal.pone.0288704 (PMC10348526; doi:10.1371/journal.pone.0288704)
Supplement: S1 File — (DOCX) [file pone.0288704.s002.docx]

###

#### INFORMATION LETTER and CONSENT FORM

**Sputum Lab Microbiology Methods for Detection of Airway Pathogens in Non-CF Bronchiectasis**

Principal Investigator: Dr. Dilini Vethanayagam Phone: (780) 492-5879

Co-Investigators: Monette Dimitrov Phone: (780) 492-6962

**Introduction**

You are being asked to be in this study because you have Non-Cystic Fibrosis (Non-CF) Bronchiectasis. Before you decide to take part of this study, you must read the following information provided on this form. Ask the study staff if you do not understand any words or information. Be sure you understand the information before signing this form. You may wish to discuss the study with your family doctor, a family member, or a close friend.

**Background and Purpose of the Research**

Non-CF Bronchiectasis is a common obstructive airways disorder. The airways become dilated or widened, and the cilia lining these airways become non-functional, which can result in difficulty clearing mucus. As a result, these features lead to a collection of thick mucus in the airways, difficult to cough up. These abnormalities lead to ongoing microbial colonization in the lower airways, which can result in constant airway infections, inflammation and a decline in lung function. Non-CF bronchiectasis can be caused by a variety disorders such as: primary ciliary dyskinesia (PCD), chronic obstructive pulmonary disease (COPD), asthma, and many others.

**Study Procedures**

To participate in this study you will answer questions regarding your condition and use of non-invasive lower airways sampling, particularly with sputum cultures (used to identify any bacterial colonizers or pathogens in your mucus). This is an interview-directed questionnaire, which will be asked by the researcher herself. The questionnaire will take approximately 10-15 minutes. Personal medical records from *Netcare* will also be accessed by the study team to retrieve the following information: sputum results from Microbiology, diagnostic imaging of the chest, and any emergency visits/hospitalizations.

This information will be collected onto a computer program that stores and organizes all data (database). The database will be made specifically for patients with Non-CF Bronchiectasis on a secured and password protected computer program. This will include patient information collected from the questionnaire and *Netcare* consisting of demographic and medical information. Demographic information collected will include: your name (which will be coded), age, and gender. Medical information we will collect include: your characteristics and symptoms of Bronchiectasis, presence of other diseases related to Non-CF Bronchiectasis, any ongoing/past treatment, whether your sputum cultures have been performed or not, and if so how they were obtained and analyzed.

This information will be compared with other participants of the same study to help analyze how sputum cultures are being performed in the Edmonton region.

**Risks of Participating in the Study**

There is a small risk that your data that has been entered into the database could be accidentally viewed by someone outside of the study. To prevent this from happening, the dataset is password protected and on computers in locked offices accessible only to the study team. We will take all available precautions to prevent this from happening.

Any identifying information will be stored separately from the data in locked cabinets within a locked room to prevent anyone outside of the study from accessing it.

**Benefits of Participating in the Study**

There are no direct benefits for participating in this study. It is hoped that the information gained from this study may improve the performance and quality of sputum cultures for patients with non-CF Bronchiectasis. This is to hopefully improve how these patients are managed and treated in the future.

**Voluntary Participation and Withdrawal**

This database is for monitoring purposes and potential research projects down the road. *You do not have to take part in this study, and if you decide not to, this will not affect your clinical care.*

If you decide to participate in this study, you may withdraw at any time. You do not need to give a reason. Please contact a member of this study team if you wish to withdraw from this study.

If you choose to withdraw from this study, your information from the database will be removed. We will not remove your information from any additional, current or completed research studies in which it has been used.

**Confidentiality & Anonymity**

All information will be held confidential, except when professional codes of ethics or legislation require reporting. Any of your personal information and records relating to this study will be kept confidential within a locked area.

The study information is required to be kept for at least 5 years. If you withdraw from the study, the medical information that is obtained from you for study purposes will not be destroyed. You have a right to check your personal and health information recorded from the questionnaire and request changes if it is incorrect.

*By signing this consent form, you are giving permission to the study staff to ask you personal and medical information about your condition of Non-CF Bronchiectasis, and to allow this information to be collected into a database so that other health care professionals can access it as deemed necessary for the research to be performed.*

*By signing this consent form, you are saying it is okay for the study team to collect, use, and disclose any information about you from your personal health records as described above.*

Contact Numbers

If you have questions about this study, please contact one of the following members of the study team on the following patient consent form attached.

If you have concerns regarding this study or your rights as a participant, you may contact the University of Alberta Research Ethics Office at (780) 492-2615. This office has no affiliation to the study or research protocol.

**Patient Consent Form**

**Non Cystic Fibrosis Bronchiectasis: Sputum Microbiology Lab Methodology and Detection of Airway Pathogens**

Principal Investigator: Dr. Dilini Vethanayagam Phone: (780) 492-5879

Co-Investigators: Monette Dimitrov Phone: (780) 492-6962

Circle One

|  |
| --- |

Do you understand that you have been asked to participate in a research study? Yes No

Have you read and received a copy of the attached Information Sheet? Yes No

Do you understand the benefits and risks involved in taking part in this study? Yes No

Do you understand that you are free to refuse to participate or withdraw from Yes No

the study at any time? You do not have to give a reason and it will not affect

your care.

Has the issue of confidentiality been explained to you? Yes No

Do you understand who will have access to your records (Netcare), including

personally identifiable health information? Yes No

*I, agree to take part in this study.*

| Printed Name of Research Participant |
| --- |

| Signature of Research Participant Date |
| --- |
| *I believe that the person signing this form understands what is involved in the study and voluntarily agrees to participate.* |
| Signature of Investigator Date |
