# Supplementary material for: Microbiology sampling in non-cystic fibrosis bronchiectasis cases from northern Alberta
Source: PLoS One. 2023 Jul 14;18(7):e0288704. doi: 10.1371/journal.pone.0288704 (PMC10348526; doi:10.1371/journal.pone.0288704)
Supplement: S2 File — (PDF) [file pone.0288704.s003.pdf]

Health Research Ethics Board

308 Campus Tower
University of Alberta, Edmonton, AB T6G 1K8
p. 780.492.9724 (Biomedical Panel)
p. 780.492.0302 (Health Panel)
p. 780.492.0459

Approval Form

Date:	July 2, 2015
Study ID:	Pro00049402
Principal Investigator:	Dilini Vethanayagam
Study Title:	Non-CF Bronchiectasis and Lower Airway Colonization: Current Methods for Sputum Microbiology Lab for Detection and Analysis of Lower Airway Pathogens
Approval Expiry Date:	Thursday, June 30, 2016
Approved Consent Form:	Approval Date: 7/2/2015 — Approved Document: Patient Information and Consent Form 2015
Sponsor/Funding Agency:	University of Alberta Faculty of Medicine and Dentistry — FOMD

Thank you for submitting the above study to the Health Research Ethics Board - Health Panel . Your application, including the following, has been reviewed and approved on behalf of the committee;

- Physician Letter for Patient Recruitment (6/23/2015)
- Clinic Coding Sheet (6/12/2015)
- Patient Questionnaires and Lab Results Non-CF Bronchiectasis (6/12/2015)
- Protocol Non-CF Bronchiectasis: Sputum Methodology Review (6/12/2015)

The Health Research Ethics Board assessed all matters required by section 50(1)(a) of the Health Information Act. Subject consent for access to identifiable health information is required for the research described in the ethics application, and appropriate procedures for such consent have been approved by the HREB Health Panel. In order to comply with the Health Information Act, a copy of the approval form is being sent to the Office of the Information and Privacy Commissioner.

A renewal report must be submitted next year prior to the expiry of this approval if your study still requires ethics approval. If you do not renew on or before the renewal expiry date (Thursday, June 30, 2016), you will have to re-submit an ethics application.

Approval by the Health Research Ethics Board does not encompass authorization to access the patients, staff or resources of Alberta Health Services or other local health care institutions for the purposes of the research. Enquiries regarding Alberta Health approval should be directed to (780) 407-6041. Enquiries regarding Covenant Health approvals should be directed to (780) 735-2274.

Sincerely,

Carol Boliek, Ph.D.
Associate Chair, Health Research Ethics Board - Health Panel

Note: This correspondence includes an electronic signature (validation and approval via an online system).

UNIVERSITY OF ALBERTA   Alberta Health Services   Covenant Health
